# Assessing Diagnostic Accuracy in Cephalometry: A Comparative Study of Manual and Digital Tracing Techniques

**DOI:** 10.7759/cureus.89412

**Published:** 2025-08-05

**Authors:** Aparajita Pandey, T P Chaturvedi, Adit Srivastava, Saumya Shukla, Savitha Priyadarsini S, Sakshee Nagvanshi, Aparna A V

**Affiliations:** 1 Orthodontics and Dentofacial Orthopaedics, Faculty of Dental Sciences, Institute of Medical Sciences, Banaras Hindu University, Varanasi, IND; 2 Oral Medicine and Radiology, Faculty of Dental Sciences, Institute of Medical Sciences, Banaras Hindu University, Varanasi, IND; 3 Public Health Dentistry, Faculty of Dental Sciences, Institute of Medical Sciences, Banaras Hindu University, Varanasi, IND

**Keywords:** artificial intelligence, cephalometry, digital analysis, manual tracing, statistical analysis

## Abstract

Aim: This study aimed to statistically evaluate and compare the accuracy, reliability, and efficiency of manual versus artificial intelligence (AI)-assisted digital cephalometric tracing using Steiner's and Down's analyses in orthodontic diagnostics.

Materials and methods: A retrospective study was conducted using 20 lateral cephalograms obtained using the NewTom GiANO HR cone-beam computed tomography (CBCT) system (Quantitative Radiology, Verona, Italy). Manual tracings were performed on acetate sheets, while digital analysis employed the AudaxCeph® software (Audax d.o.o., Ljubljana, Slovenia) with automated landmark detection. Measurements were analysed using independent sample t-tests and Mann-Whitney U tests, with significance set at p ≤ 0.05.

Results: Statistical analysis showed no significant differences (p > 0.05) between manual and digital cephalometric measurements across key parameters. Minor variations were within clinically acceptable limits, confirming that both methods are consistent and interchangeable for orthodontic diagnostics.

Conclusion: AI-assisted digital cephalometric tracing is as accurate and reliable as manual methods, offering enhanced efficiency and consistency. These findings support its integration into routine orthodontic diagnostics.

## Introduction

Cephalometrics has long been an integral part of orthodontics, playing an essential role in diagnosis, treatment planning, and post-treatment evaluation. It is instrumental in assessing dental and skeletal relationships, monitoring craniofacial growth, and refining orthodontic techniques to improve clinical outcomes. Additionally, cephalometric analysis supports research and enhances the predictability of orthodontic interventions [1,2].

Cephalometric tracing can be performed using either manual or digital methods. Manual tracing involves identifying anatomical landmarks on radiographic films using acetate overlays. These landmarks serve as reference points to draw planes and lines and measure linear and angular dimensions. A millimeter scale and protractor are commonly used to obtain precise measurements in manual analyses [3]. However, manual cephalometry is time-consuming and inherently subject to human error. Inter-operator variability, difficulty in storing physical tracings, and the degradation of acetate sheets over time further compromise accuracy and reproducibility [4].

With the rapid evolution of digital dentistry, digital cephalometry has emerged as a more efficient and accessible alternative. It offers several advantages over manual tracing, including reduced analysis time, enhanced landmark visibility through image processing, and simplified data storage. However, digital cephalometry is not without limitations. Potential drawbacks include eye strain, screen resolution issues, and the need for operator training and experience [5].

The integration of artificial intelligence (AI) into digital cephalometric systems has brought about further innovation, offering promising improvements in diagnostic accuracy and workflow efficiency. AI-driven systems minimize human error, automate landmark detection and measurements, and reduce measurement bias. Nevertheless, their accuracy can still be affected by factors such as image artifacts, improper calibration, and variability in algorithm design across different software platforms [6].

The advancement of cephalometric software compatible with multiple platforms (Windows, iOS, Android) has made cephalometry more user-friendly and accessible for clinicians. These systems now allow the automated detection of cephalometric landmarks and real-time calculation of linear and angular measurements. Despite these developments, accurate landmark identification remains the foundation of reliable cephalometric analysis.

A comparative study done by Albarakati et al. examined the reliability of manual versus digital cephalometric methods and concluded that both methods yielded statistically equivalent results across all landmarks and measurements [7]. However, digital methods demonstrated greater efficiency and consistency, particularly for angular measurements. The study emphasized the need for operator training regardless of the modality used [8].

Till date, only a limited number of studies have directly compared the accuracy and reliability of manual versus digital cephalometric analysis. Therefore, this original study was conducted to evaluate and compare cephalometric measurements obtained through manual tracing and digital software tracing, utilizing Steiner's analysis and Down's analysis.

## Materials and methods

Study design

The present study was a retrospective study. The data was collected from archived patient records and lateral cephalometric imaging data obtained from the Department of Oral Medicine and Radiology, Faculty of Dental Sciences, Institute of Medical Sciences, Banaras Hindu University, Varanasi, India, from January 2025 to April 2025. The study was approved by the Institutional Ethics Committee of the Institute of Medical Sciences, Banaras Hindu University, on October 9, 2023 (approval number: Dean/2023/EC/6687). The study adhered to the principles outlined in the Declaration of Helsinki.

Sample size estimation

Sample size was calculated based on the results of the study by Kamath and Arun using the G*Power software (Ver. 3.1.9.7 Heinrich-Heine-Universität Düsseldorf, Düsseldorf, Germany) with an effect size of 1.147, a study power of 95%, and an alpha error of 0.05 [4]. The minimum required samples were 20 per group.

Study population

**Figure 1:**
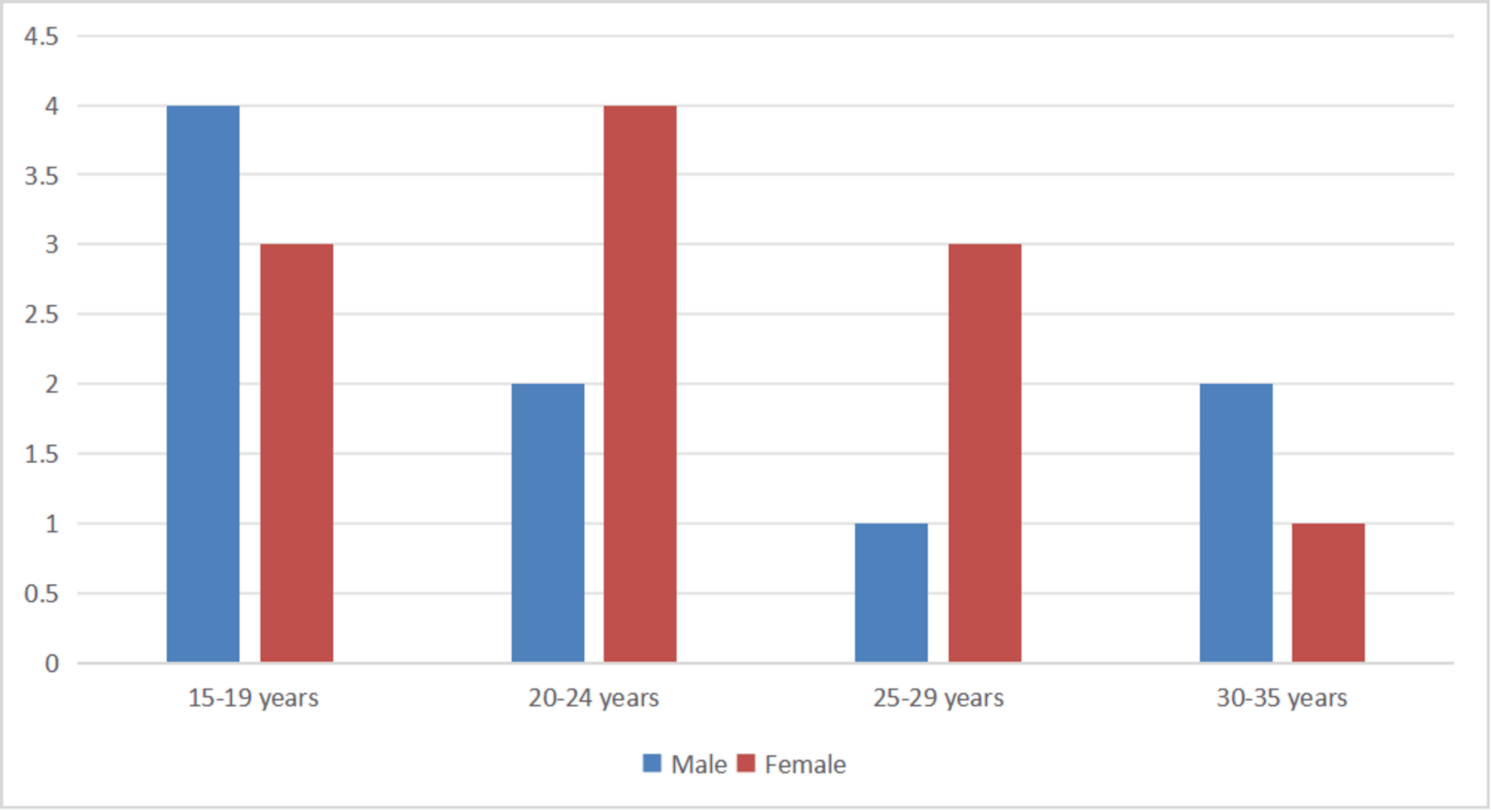
Demographic chart of the study population

Archived lateral cephalograms of patients aged 15-35 years including both male and female individuals were retrieved and reviewed. The records were then selected based on the relevance of the predefined inclusion criteria. The criteria included high-quality lateral cephalograms of patients with no history of craniofacial anomalies, prior orthodontic treatment, trauma, or orthognathic surgery. Exclusion criteria comprised incomplete records, poor image quality, or any prior orthodontic intervention that could affect craniofacial morphology (Figure 1).

Intraclass correlation coefficient (ICC)

A single examiner was trained for digital assessment, and intra-examiner reliability was assessed using an ICC on 10 lateral cephalograms which were not included in the main study. The value of ICC was 0.83, which indicated almost perfect agreement. The manual analysis was performed twice by the same orthodontist on 10 lateral cephalograms with a time interval of two weeks, and the value of ICC was found to be 0.81, which indicated almost perfect agreement.

Imaging protocol

All lateral cephalograms were acquired using the NewTom GiANO HR cone-beam computed tomography (CBCT) system (Quantitative Radiology, Verona, Italy) equipped with a dedicated cephalometric arm. The imaging protocol was standardized using preset parameters, namely, tube voltage: 90 kVp, tube current: 10 mA, and exposure time: ~3.5 seconds.

Manual cephalometric tracing

Manual tracings were carried out by a single orthodontic specialist using standardized methods. Each tracing was performed on 8" × 10" radiographic film prints placed over a view-box (Dentaurum, Ispringen, Germany) in a dimly lit room to enhance landmark visibility. Transparent acetate tracing sheets (8" × 10") were fixed over the radiographs using adhesive tape. A 0.5 mm mechanical pencil was used to mark key anatomical landmarks: S (sella), N (nasion), A (subspinale), B (supramentale), Gn (gnathion), and Go (gonion), Or (orbitale), Po (porion), and Me (menton). Also, various landmarks relevant to the study objectives were precisely located. SN (sella-nasion) plane and Frankfort horizontal plane (orbitale-porion) were drawn for the reference plane of Steiner's analysis and Down's analysis, respectively (Table 1 and Table 2).

**Table 1:** Cephalometric parameters and abbreviations in Steiner's analysis

S. no.	Parameter	Abbreviation	Norm value	Unit
1	Sella-nasion to A point	SNA	82° ± 2°	Degrees
2	Sella-nasion to B point	SNB	80° ± 2°	Degrees
3	A point to B point	ANB	2° ± 2°	Degrees
4	Occlusal plane to SN	Occl-SN	14° ± 2°	Degrees
5	Mandibular plane to SN	MP-SN	32° ± 5°	Degrees
6	Upper incisor to NA (angular)	U1-NA	22° ± 2°	Degrees
7	Upper incisor to NA (linear)	U1-NA (mm)	4 mm ± 2 mm	Millimeters
8	Lower incisor to NB (angular)	L1-NB	25° ± 2°	Degrees
9	Lower incisor to NB (linear)	L1-NB (mm)	4 mm ± 1 mm	Millimeters
10	Upper lip to S line	UL-S line	0 mm ± 2 mm	Millimeters
11	Lower lip to S line	LL-S line	0 mm ± 2 mm	Millimeters

**Table 2:** Summary of cephalometric measurements and standard abbreviations in Down's analysis

S. no.	Parameter	Abbreviation	Norm value	Unit
1	Facial angle	FA	87.8° ± 3.6°	Degrees
2	Angle of convexity (A-N-Pg)	Angle of convexity	0° ± 5.5°	Degrees
3	A-B plane angle	AB plane	-4.6° ± 4.2°	Degrees
4	Mandibular plane angle (MP-FH)	MP-FH	21.9° ± 4°	Degrees
5	Y-axis angle (SGn-FH)	Y-axis	59.4° ± 3.8°	Degrees
6	Cant of occlusal plane (Occl-FH)	Occl-FH	9.3° ± 3.4°	Degrees
7	Interincisal angle	U1-L1	135.4° ± 6.4°	Degrees
8	Incisor occlusal plane angle (L1-Occl)	L1-Occl	55.8° ± 4.5°	Degrees
9	Lower incisor mandibular plane angle	L1-MP	90.1° ± 5.7°	Degrees
10	Upper incisor to A-Pog line	U1 to A-Pog	2 mm ± 1 mm	Millimeters

**Figure 2:**
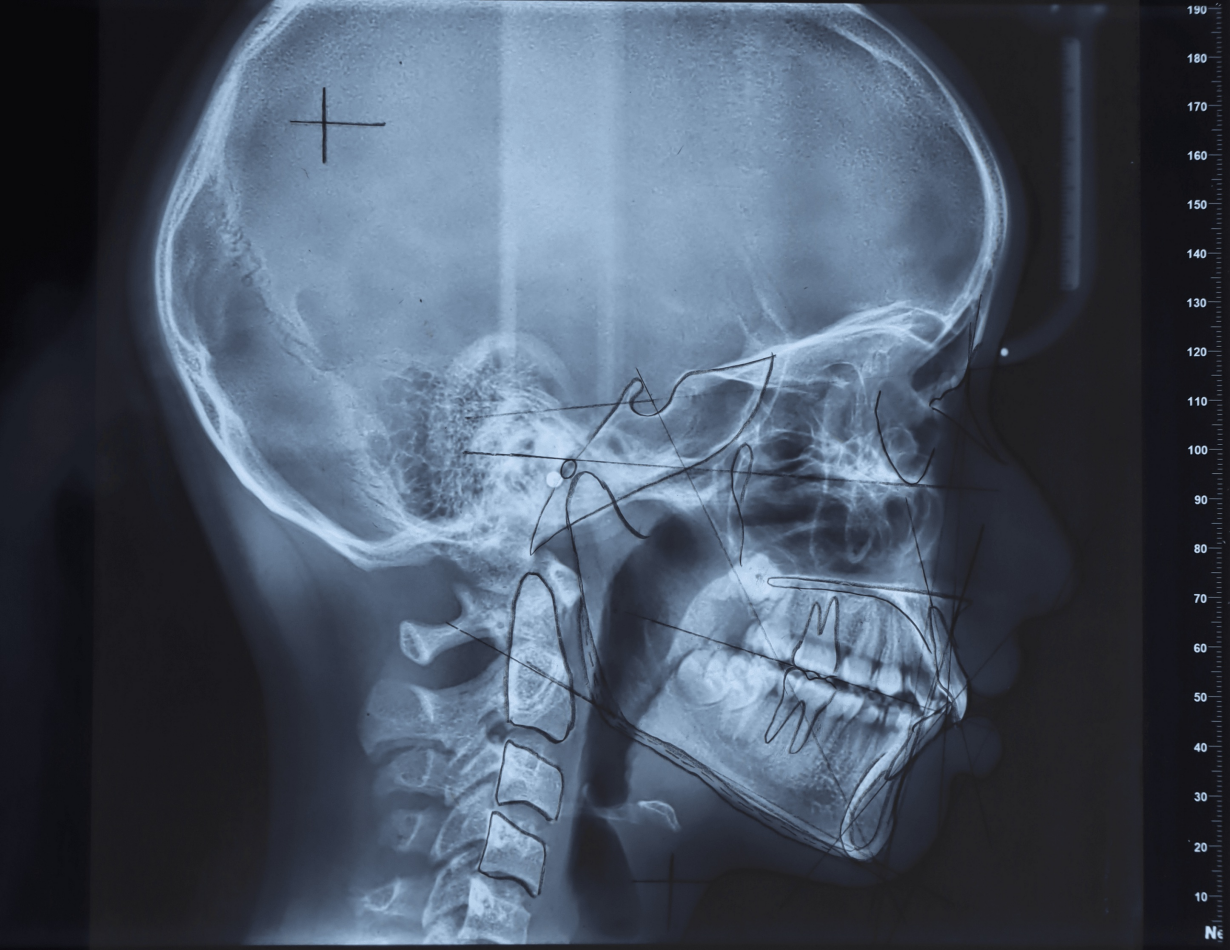
Manual cephalometric tracing

Measurements were performed using a ruler with an integrated protractor (Figure 2). All tracings and analysis were conducted by the same examiner to eliminate inter-observer variability.

Digital cephalometric analysis

**Figure 3:**
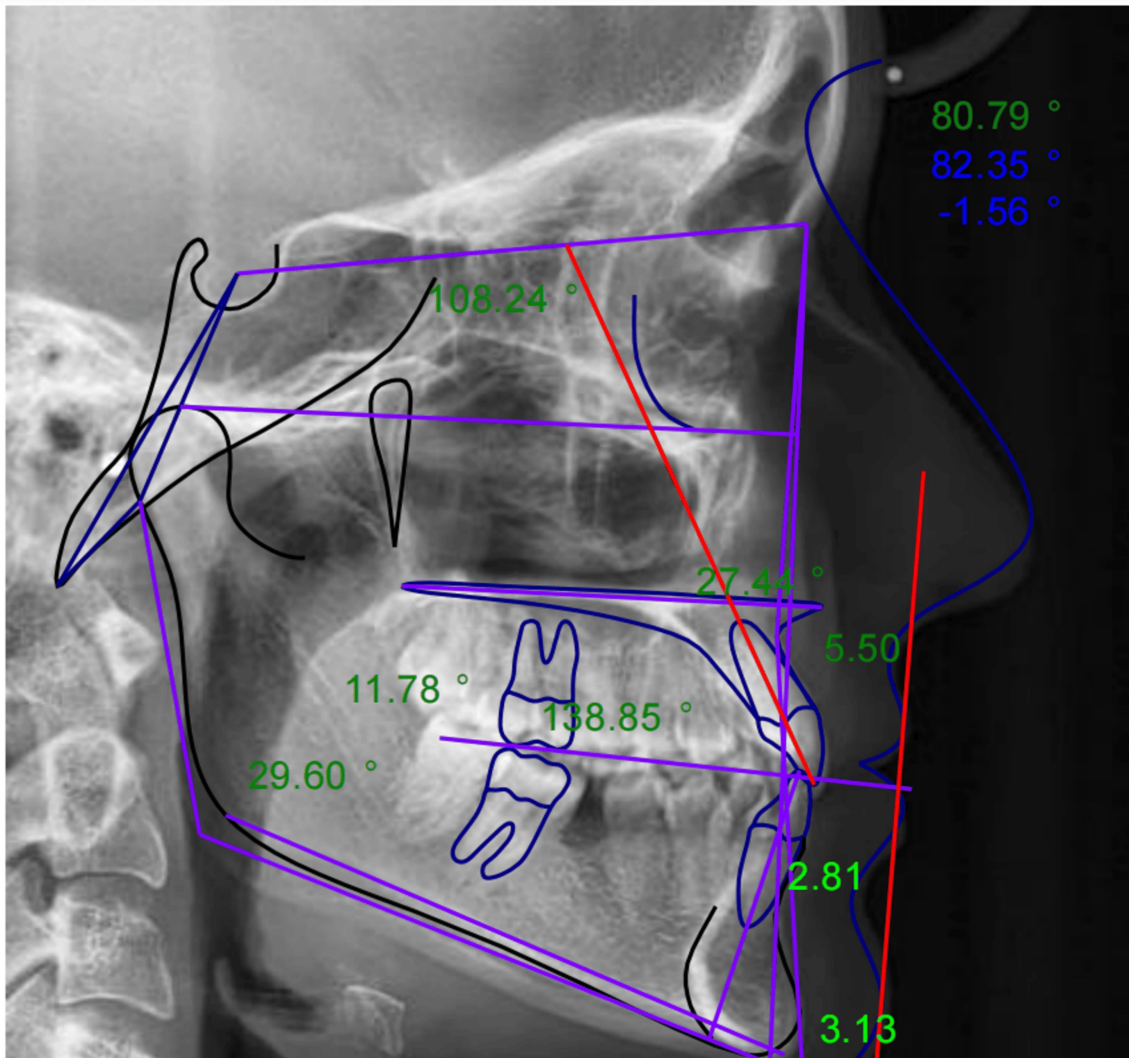
Digital Steiner's analysis using the AudaxCeph® software

Digital cephalometric analysis was performed using the AudaxCeph® software version 6.4.18.4523 (Audax d.o.o., Ljubljana, Slovenia) by an experienced oral radiologist. The software employs AI to detect craniofacial landmarks automatically. No manual modifications were made to the AI-generated landmarks. Each image was calibrated using the software's built-in digital ruler to ensure precise scaling. Digital measurements included all parameters outlined in Steiner's and Down's analyses and were generated automatically by the software (Figure 3).

Data compilation and statistical analysis

The data was compiled in Microsoft Excel 2021 (Microsoft Corp., Redmond, WA, USA) and analysed using IBM SPSS Statistics for Windows, V. 25.0 (IBM Corp., Armonk, NY, USA). Normality was assessed using the Shapiro-Wilk test. The data of variables (SNA, SNB, MP-SN, U1-NA, U1-NA (mm), L1-NB, LL-S line, facial angle, MPa, Y-axis, C of OCC, U1-L1, L1 to OCC, L1 to Mand, U1 to A Pog) was found to be normally distributed; hence, a parametric test, that is, independent sample t-test, was used for intergroup analysis. The data of variables (ANB, OC-SN, L1-NB (mm), UL-S line, angle of convexity, A-B plane angle) were not normally distributed; hence, a non-parametric test, that is, Mann-Whitney U test, was used for intergroup analysis. A p-value of ≤0.05 was considered statistically significant.

## Results

This study involved a comparative evaluation of cephalometric parameters obtained through two measurement techniques, manual tracing (group 1) and digital analysis (group 2), assessed using Steiner's and Down's analyses.

Descriptive analysis revealed generally comparable mean values between manual and digital techniques for most variables. For instance, the SNA angle was 84.07 ± 4.78 (manual) vs. 82.87 ± 5.43 (digital); SNB was 80.80 ± 4.03 (manual) vs. 78.87 ± 5.24 (digital); and ANB was 3.27 ± 3.49 (manual) vs. 4.07 ± 3.21 (digital). For linear values, U1-NA were 6.40 ± 4.06 mm (manual) vs. 5.33 ± 3.08 mm (digital), and L1-NB was 10.30 ± 18.52 mm (manual) vs. 10.60 ± 17.86 mm (digital), respectively.

Table 3 and Table 4 show the intergroup comparison of cephalometric parameters, where there were no statistically significant differences (p > 0.05) between manual and digital methods, indicating measurement consistency, for example, SNA (p = 0.293), SNB (p = 0.162), MP-SN (p = 0.936), U1-NA (p = 0.435), and L1-MP (p = 0.958). The Mann-Whitney U test also showed non-significance for ANB (p = 0.436), OC-SN (p = 0.713), upper lip to S-line (p = 0.967), A-B plane angle (p = 0.744), and angle of convexity (p = 0.595). Homogeneity of variance between the groups was verified using Levene's test prior to applying the t-tests. The test confirmed that variance between the groups was statistically equal, satisfying a key assumption of the t-test analysis.

**Table 3:** Intergroup comparison of SNA, SNB, MP-SN, U1-NA, U1-NA (mm), L1-NB, LL-S line, facial angle, MPa, Y-axis, C of OCC, U1-L1, L1 to OCC, L1 to Mand, and U1 to A Pog between manual and digital analyses *Independent sample t-test is used; p ≤ 0.05 is considered statistically significant. S.A. SNA: Steiner's analysis-sella-nasion to A point; S.A. SNB: Steiner's analysis-sella-nasion to B point; S.A. MP-SN: Steiner's analysis-mandibular plane to SN; D.A. U1-NA: Down's analysis-upper incisor to NA; D.A. U1-NA (mm): Down's analysis-upper incisor to NA (mm); D.A. L1-NB: Down's analysis-lower incisor to NB; LL-S line: lower lip to S line; MPa: mandibular plane angle; C of OCC: cant of occlusal plane; U1-L1: upper incisor to lower incisor; L1 to OCC: lower incisor to occlusal plane; L1 to Mand: lower incisor to mandibular plane; U1 to A Pog: upper incisor to A-pogonion line

Variables	Manual (n = 20)	Digital (n = 20)	t-value	P-value*
S.A. SNA	84.07 ± 4.78	82.87 ± 5.43	0.64	0.29
S.A. SNB	80.80 ± 4.03	78.87 ± 5.24	1.13	0.16
S.A. MP-SN	27.60 ± 4.89	30.87 ± 5.26	-1.76	0.93
D.A. U1-NA	27.47 ± 8.90	24.73 ± 8.42	0.86	0.43
D.A. U1-NA (mm)	6.40 ± 4.06	5.33 ± 3.08	0.80	0.18
D.A. L1-NB	27.47 ± 8.40	27.07 ± 8.49	0.13	0.95
LL-S line	3.00 ± 3.16	2.67 ± 3.10	0.29	1.00
Facial angle	87.67 ± 3.06	87.67 ± 4.53	0.00	0.05
MPa	23.13 ± 5.26	22.27 ± 5.40	0.44	0.48
Y-axis	59.20 ± 4.26	59.93 ± 3.63	-0.50	0.54
C of OCC	7.47 ± 3.18	5.27 ± 4.72	1.49	0.20
U1-L1	123.73 ± 12.47	120.87 ± 12.02	0.64	0.66
L1 to OCC	25.20 ± 7.21	27.27 ± 7.07	-0.79	0.77
L1 to Mand	9.67 ± 7.64	10.40 ± 7.61	-0.26	0.72
U1 to A Pog	8.20 ± 2.78	8.27 ± 3.32	-0.06	0.30

**Table 4:** Intergroup comparison of S.A. ANB, S.A. OC-SN, L1-NB (mm), UL-S line, Angle of Con, and A-B plane between manual and digital analyses *Mann-Whitney U test is used; p≤ 0.05 is considered statistically significant. S.A. ANB: Steiner's analysis-A point to B point; S.A. OC-SN: Steiner's analysis-occlusal plane to SN; L1-NB (mm): lower incisor to NB (mm); UL-S line: upper lip to S line; Angle of Con: angle of convexity; A-B plane: A point to B point plane

Variables	Manual	Digital	Mean rank	Z-value	P-value*
S.A. ANB	3.27 ± 3.49	4.07 ± 3.21	14.20	-0.81	0.43
S.A. OC-SN	13.60 ± 5.71	17.87 ± 17.51	14.90	-0.37	0.71
L1-NB (mm)	10.30 ± 18.52	10.60 ± 17.86	15.03	-0.29	0.77
UL-S line	2.73 ± 3.39	2.80 ± 3.09	15.40	-0.06	0.96
Angle of Con	4.80 ± 8.97	6.40 ± 8.15	14.63	-0.54	0.59
A-B plane	-8.07 ± 8.73	-8.73 ± 9.41	16.07	-0.35	0.74

## Discussion

The current study sought to evaluate and compare cephalometric measurements obtained via manual tracing and digital analysis, two widely used methods in orthodontic diagnostics. A total of 20 cases per group were analysed across multiple angular and linear parameters, offering a robust dataset for comparison.

The statistical analysis, using independent sample t-tests for normally distributed data (Table 3) and Mann-Whitney U tests for non-normally distributed data (Table 4), revealed no statistically significant differences between manual and digital methods across any of the evaluated parameters. This suggests that both methods yield comparable cephalometric values. The facial angle (p = 0.054) was the only variable that approached statistical significance, indicating a potential area for further investigation in studies with larger sample sizes to increase statistical power.

The results are well-supported by prior investigations. Studies such as those by Santoro et al. and Moshiri et al. have found that digital tracings demonstrate excellent agreement with manual analyses, especially when landmark identification is consistent and software tools are calibrated accurately [8,9], while minimal discrepancies are occasionally reported, especially in soft tissue measurements. Additionally, the study by Agrawal et al. compared the reproducibility of digital cephalometric analysis with traditional methods. They concluded that digital systems offer significant time efficiency and ease of use, without compromising measurement accuracy [10]. Albarakati et al. demonstrated comparable reproducibility across conventional and digital methods, highlighting consistency in both linear and angular cephalometric landmarks [7]. A study by Khan et al. found no statistically significant discrepancies in 95% of evaluated measurements [11]. Azeez et al. further validated newer platforms like WebCeph™, confirming that web-based digital systems can reliably substitute manual methods [12]. More recent systematic reviews by Narkhede et al. and Sadek et al. extend the conversation towards automation and AI integration [13,14]. These studies emphasize that with proper training and calibration, digital tools, including those that use AI for landmark detection, are not only accurate but also time-efficient and useful for remote collaboration, especially in teledentistry settings [15].

The results of this study suggest that both manual and digital cephalometric analyses yield comparable outcomes, reinforcing the clinical interchangeability of these techniques. The benefits of digital systems include reduced tracing time, efficiency, consistency, and ease of storage. It can be used in daily practice without sacrificing accuracy, especially useful in high-volume practices where time-saving tools are essential. Additionally, the foundational skills of manual tracing remain vital in clinical education and should be preserved as a benchmark for evaluating digital results. Ultimately, these findings encourage us for a progressive shift towards digital analysis. These findings validate the routine clinical use of digital cephalometry while emphasizing continued attention to training and standardization.

Limitations

A larger sample size is needed for increased statistical power for further exploration. The accuracy of both manual and digital cephalometric analysis relies on the quality and resolution of the initial radiographic images. Poor image quality could introduce measurement errors regardless of the analysis method. Moreover, there is a valid concern that inadequately trained individuals may misinterpret radiographic data, irrespective of the method used, resulting in diagnostic errors and improper patient care.

## Conclusions

Digital cephalometric tracing has proven to be a reliable and valid alternative to traditional manual techniques. With proper calibration, especially in systems enhanced by AI, digital tools can match manual methods in both accuracy and reproducibility. 
